# Editorial: Reviews in recommender systems: 2022

**DOI:** 10.3389/fdata.2024.1384460

**Published:** 2024-04-02

**Authors:** Dominik Kowald, Deqing Yang, Emanuel Lacic

**Affiliations:** ^1^Know-Center GmbH, Graz, Austria; ^2^Institute of Interactive Systems and Data Science, Graz University of Technology, Graz, Austria; ^3^School of Data Science, Fudan University, Shanghai, China; ^4^Infobip, Zagreb, Croatia

**Keywords:** recommender systems, review, fairness, privacy, collaborative filtering

## 1 Introduction

Nowadays, recommender systems are one of the most widely used instantiations of machine learning and artificial intelligence. Thus, these systems accompany us in our daily online experience and have become an integral part of our digital life for supporting us in finding relevant information in information spaces that are too big or complex for manual filtering (Ricci et al., 2010; Burke et al., 2011; Jannach et al., 2016). Since the first deployments of recommendation algorithms (Resnick et al., 1994; Resnick and Varian, 1997), recommender systems analyze past usage behavior (e.g., clicks or ratings) in order to build user models, and to suggest items to users. Recommender systems are employed in various domains, ranging from entertainment domains, such as music (Lex et al., 2020; Schedl et al., 2021) and movies (Harper and Konstan, 2015), to more critical domains such as the job market (Lacic et al., 2020). Apart from that, different types of algorithms have been employed to develop recommender systems, ranging from collaborative filtering (Ekstrand et al., 2011), content-based filtering (Lops et al., 2010), hybrid approaches (Burke, 2002), theory-driven algorithms [e.g., based on cognitive models (Lacic et al., 2014; Kowald et al., 2015)], to neural approaches (Zhang et al., 2019; Chen et al., 2023).

The aim of the “*Reviews in recommender systems*” Research Topic is to highlight recent advances in the broad field of recommender systems, including important topics such as fairness (Kowald et al., 2020; Wang et al., 2023), privacy (Friedman et al., 2015; Muellner et al., 2021), and multi-stakeholder objectives (Abdollahpouri and Burke, 2019), while emphasizing novel directions and possibilities for future research. In total, this Research Topic consists of nine review articles surveying the literature in a specific subfield of recommender systems. More concretely, the editors of this Research Topic have been able to accept six full-length and three mini review articles. The following section gives a short overview of these articles.

## 2 Research Topic content

In a mini review article, Müllner et al. surveyed the current landscape of differential privacy in collaborative filtering-based recommender systems. In total, the authors have reviewed 26 publications, and found that in most cases, differential privacy is applied to the user representation (i.e., the input data of the recommender system) rather than to recommendation model updates or to phases after the training. Additionally, the authors stated that most papers investigate differential privacy on datasets gathered from MovieLens and Last.fm, and thus, that more research is needed for privacy-aware recommender systems in sensitive domains such as the job market or finance. Next, Jannach and Abdollahpouri explore the multifaceted landscape of multi-objective recommender systems, identifying the need to balance diverse and often conflicting objectives such as user satisfaction, stakeholder interests, and long-term goals of stakeholders. The authors present a taxonomy categorizing these objectives into recommendation quality, multi-stakeholder perspectives, temporal considerations, user experience, and system engineering challenges. The study illustrates the complexity of optimizing recommender systems in real-world applications, emphasizing the importance of addressing multiple objectives to enhance recommendation relevance, diversity, and overall system effectiveness.

Banerjee et al. delve into the challenges and potential strategies for ensuring fairness in Tourism Recommender Systems (TRS), emphasizing the multi-stakeholder nature of these systems. They categorize stakeholders based on fairness criteria, review state-of-the-art research from various perspectives, and highlight the complexities of balancing individual and collective interests. The paper concludes that achieving fairness in TRS involves navigating trade-offs between stakeholder interests, illustrating the necessity for innovative solutions that consider the environmental impact and societal concerns alongside traditional user and provider objectives. In the next mini-review, Loepp investigates the increasingly prevalent multi-list user interfaces in recommender systems, particularly focusing on carousel-based interfaces like those used by Netflix and Spotify. The review highlights the scarcity of research on optimizing these carousels for user interaction and satisfaction, despite their common use. Based on 18 reviewed research papers, the author identifies gaps in understanding user behavior and interface design, and proposes future research directions to enhance user experience through improved design and personalization of carousel recommendations.

Kumar et al. provide an in-depth review of fairness in recruitment-related recommender systems (RRSs), dissecting the balance between technical advancements and legal compliance. They delve into various fairness definitions (e.g., demographic parity), metrics (e.g., false positive rates between different demographic groups), and debiasing strategies (e.g., post-processing to alter the algorithm's output to ensure fairness) as well as compare them to existing EU and US employment laws. The survey spotlights the nuanced challenges of mitigating algorithmic bias and discrimination within RRSs, advocating for a multidisciplinary approach to develop more equitable and legally compliant hiring technologies. Additionally, Felfernig et al. explore the potential of recommender systems to support the achievement of the 17 United Nations' Sustainability Development Goals (SDGs). The review addresses the utilization of AI to recommend actions and alternatives aligned with sustainability objectives. The paper discusses various recommender system types, their application across all SDGs, as well as identifies open research issues for future exploration. The authors show the significance of recommender systems in promoting sustainability, offering both current insights and directions for ongoing research.

In this mini-review, Duricic et al. explore the integration of beyond-accuracy metrics (i.e., diversity, serendipity, and fairness) into recommender systems based on Graph Neural Networks (GNNs). They emphasize the importance of these metrics in enhancing user satisfaction, beyond mere accuracy. Furthermore, they examine recent advancements and methodologies in GNNs that address these dimensions, highlighting the balance between recommendation accuracy and beyond-accuracy objectives. Next, Lubos et al. present a review of state-of-the-art video recommender systems (VRS), covering a broad range of algorithms, applications, and unresolved research challenges in the field. They delve into various approaches to VRS, including content-based, collaborative filtering, and hybrid systems, and discuss the importance of diverse content representations and evaluation metrics. Based on the analysis of 6 different application domains, they highlight the potential for future advancements in VRS, emphasizing the need for innovative solutions to improve the accuracy and effectiveness of personalized video recommendations, thereby serving as a valuable resource for both researchers and practitioners in the video domain. Finally, Uta et al. offer a comprehensive overview of knowledge-based recommender systems, distinguishing them from traditional collaborative and content-based approaches by their ability to utilize semantic user preferences, item knowledge, and recommendation logic. These systems are particularly beneficial for complex item types, as they can dynamically adapt to user preferences through dialogue and constraint-based recommendations. The review also identifies future research directions, emphasizing the integration of knowledge-based technologies in recommender systems.

Taken together, across all review articles, we see that beyond-accuracy objectives and trustworthiness aspects of recommender systems are currently of high interest in the recommender systems research community. This includes aspects related to fairness, bias, privacy, diversity, serendipity, sustainability, multi-stakeholder objectives, and user interface choices. We hope that the review articles presented in this Research Topic will inform future research endeavors in this field.

## Author contributions

DK: Writing – original draft, Writing – review & editing. DY: Writing – original draft, Writing – review & editing. EL: Writing – original draft, Writing – review & editing.
